# Supplementary material for: miRNA Library Preparation Optimisation for Low-Concentration and Low-Volume Paediatric Plasma Samples
Source: Noncoding RNA. 2025 Feb 5;11(1):11. doi: 10.3390/ncrna11010011 (PMC11858269; doi:10.3390/ncrna11010011)
Supplement: Supplementary file 1 [file ncrna-11-00011-s001.zip › ncrna-3372385-supplementary.pdf]

Supplementary Materials

Optimised QIAseq miRNA Library Preparation Protocol for Low Concentration and Low Volume Biofluids

Modified from the QIAseq miRNA UDI Library Kit Handbook for Illumina NGS using Unique Dual Indexes

Qiagen. QIAseq® miRNA UDI Library Kit Handbook Precision small RNA library prep for Illumina® NGS systems using unique dual indexes (UDIs) [Internet]. Hilden, Germany: Qiagen, 2023. [cited 2024 November 21]. 52 p. [08/2023]. Available from: https://www.qiagen.com/us/products/discovery-and-translational-research/next-generation-sequencing/rna-sequencing/mirna-small-rnaseq/qiaseq-mirna-ngs

Supplementary Materials S1 **Prior sample preparation**

100µl of starting plasma was extracted with the miRNeasy total RNA Extraction Kit (Qiagen), 12µl of total RNA elute will be the final product. 1.2µl of this total RNA elute was used for producing cDNA to check for inhibitors via qPCR with probes for UniSp6. High Ct values for UniSp6 denotes the presence of high levels of inhibitors in the sample. The remaining 10.8µl total RNA elute was condensed until dry with the Savant SPD1010 SpeedVac Concentrator (ThermoScientific) drying temperature was 45 degrees Celsius; drying pressure was set to 5.1 Torr (observed pressure was between 6 and 10 Torr). The total RNA was then resuspended in 5µl of RNase free water.

Day 1

Supplementary Materials S2 **3' Ligation**

- Do not exceed 12 samples per batch.
- Ensure the starting material has been condensed from 10.8µl to 5µl.
- Set up all reactions on ice.
- Reactions are very viscous, to mix, pipette slowly 20 times.
- Do not vortex NGS RI, 3' Ligase, template RNA or 3' Ligation reactions.
- Ensure all reagents are added in the order stated in reaction mix tables.

1. Thaw the condensed template RNA, mix by flicking tube and briefly centrifuge. Keep on ice until ready.

2. Thaw QIAseq miRNA NGS 3' adaptor, NGS 3' buffer, 2x ligation activator and nuclease free water. Mix by flicking and briefly centrifuge.
3. Dilute the QIAseq miRNA NGS 3' adaptor **1:20** (eg. 1µl 3' adaptor to 19µl nuclease free water). Mix 20 times, and briefly centrifuge.
4. Remove QIAseq miRNA NGS RI and 3' ligase from the freezer just before use and keep on ice. Return to freezer immediately after use.
5. Prepare the 3' ligation reaction on a 96 well PCR plate on ice:

Reagent	***Volume (µl)***
QIAseq miRNA 3' adaptor	1
QIAseq miRNA RI	1
QIAseq miRNA 3' ligase	1
QIAseq miRNA 3' buffer	2
Ligation activator (*viscous*)	10
RNA template	5
Total Volume	***20***

Table S1. Preparation of the 3' Ligation reaction mix. Please add each reagent in the order specified and ensure the 3' adaptor is diluted 1:20.

6. Seal the plate loosely, and briefly centrifuge. Then mix the reaction by pipetting 20 times, reseal completely and briefly centrifuge again.
7. Incubate the plate on the thermocycler as follows:

**Temperature (°C)**	**Time (minutes)**
**28**	60
**65**	20
**4**	10

Table S2. Thermocycler incubation for 3' ligation reactions.

Proceed immediately to the 5' Ligation Protocol

Supplementary Materials S3 **5' Ligation**

- Ensure the **UDI** 5' adaptor is used (not the NGS 5' adaptor), found in the UDI Index Kit.
- The entire 20µl 3' ligation reaction from the previous step is the starting material. Add to the same plate.
- Set up all reactions on ice.
- Do not vortex the NGS RI, UDI 5' adaptor.
- Ensure all reagents are added in the order stated in reaction mix tables.

1. Thaw the UDI 5' adaptor and the NGS buffer at room temperature. Mix by flicking the tubes and briefly centrifuge. Keep at room temperature.
2. Dilute the UDI 5' adaptor **1:10** (eg. 2µl adaptor to 18µl nuclease free water). Mix by pipetting 20 times and briefly centrifuge.
3. Remove the NGS RI and the 5' ligase from the freezer. Keep on ice and return to freezer immediately after use.
4. Prepare the 5' ligation reaction as follows:

Reagent	***Volume (µl)***
3' ligation reaction (*already in wells*)	***20***
Nuclease free water	***15***
QIAseq miRNA 5' buffer	***2***
QIAseq miRNA RI	***1***
QIAseq miRNA 5' ligase	***1***
UDI 5' adaptor (*diluted*)	***1***
Total Volume	***40***

Table S3. Preparation of the 5' Ligation reaction mix. Please add each reagent in the order specified and ensure the UDI 5' adaptor is diluted 1:10.

5. Incubate in the thermocycler as follows:

**Temperature (ºC)**	**Time (minutes)**
**28**	30
**65**	20
**4**	10

Table S4. Thermocycler incubation for 5' ligation reactions.

Make up QMN Beads at this stage

Supplementary Materials S4 **Preparation of QIAseq miRNA Beads (QMN Beads)**

- The QMN beads need to be homogeneous, this means working quickly, and in case of any settling of the beads, vortex to ensure they are mixed well.

- After preparation, the beads must be kept on ice.

1. Thoroughly vortex the QMN beads, do not centrifuge.
2. Carefully add 400µl of the QMN beads to a 2ml microfuge tube. Briefly centrifuge and immediately separate on the magnetic stand.
    - Note: 400µl is enough for 1 sample. Times 400 * sample number to get the total volume required (eg: 400* 4 = 1600).
3. When the beads are fully migrated, carefully discard the supernatant (it's ok to leave a small amount of supernatant in the tube at this stage).
4. Remove from the magnetic stand and carefully pipette (buffer is viscous) 150µl of the bead binding buffer onto the beads. Remember to times by the sample number (eg: 4* 150 = 600µl).
    - Thoroughly vortex, briefly centrifuge and immediately separate the beads on the magnetic stand.
5. When the beads have fully migrated, carefully discard as much of the supernatant as possible.
6. Remove from the magnetic stand and add 400µl of the bead binding buffer onto the beads and thoroughly vortex.
    - Again, times the 400µl by the sample number (eg: 400*4 = 1600µl).

Keep on ice/ in fridge until use.

QMN beads can be stored at 2-8°c for up to a week.

Supplementary Materials S5 **Reverse Transcription**

- The entire 40µl from the previous reaction is the starting material.
- Set up reactions on ice.
- Do not vortex reagents.
- Ensure to use the **UDI** RT initiator (found in the UDI Index Kit).
- Ensure all reagents are added in the order stated in reaction mix tables.

1. Thaw the UDI RT initiator, mix by flicking tubes and briefly centrifuge.
2. Add 2µl of the UDI RT initiator to each well. Seal plate and briefly centrifuge, mix by pipetting 20 times, seal and briefly centrifuge again.

3. Incubate plate in the thermocycler as follows (volume = 42µl):

Temperature (ºC)	Time (minutes)
**75**	2
**70**	2
**65**	2
**60**	2
**55**	2
**37**	5
**25**	5
**4**	∞

Hold until the setup of the RT reaction

Table S5. Thermocycler incubation for UDI RT initiator reactions.

4. Dilute the RT primer **1:20** (eg. 2µl RT primer in 38µl nuclease free water).
5. On ice, prepare the RT reaction as follows:

Reagent	***Volume (µl)***
5' Ligation reaction + RT initiator	42
QIAseq miRNA RT primer (diluted)	2
Nuclease free water	2
QIAseq miRNA RT buffer	12
QIAseq miRNA RI	1
QIAseq miRNA RT enzyme	1
Total Volume	***60***

Table S6. Preparation of the RT reaction mix. Please add each reagent in the order specified and ensure the RT primer is diluted 1:20.

6. Seal plate and briefly centrifuge. Mix by pipetting 20 times, seal and briefly centrifuge again.
7. Incubate plate in the thermocycler as follows:

Temperature (ºC)	Time (minutes)
**50**	60
**70**	15
**4**	10

Table S7. Thermocycler incubation for RT reactions.

Move to PCR hood at this stage

Supplementary Materials S6 **cDNA Clean Up**

- The entire 60µl reaction from the RT protocol is the starting material.
- The QMN beads are required.
- Prepare FRESH 80% ethanol with nuclease free water
- Following all ethanol washes, beads must be completely dry.

1. Transfer the 60µl from the 96 well plate to labelled 1.5ml tubes.
2. Ensure the QMN beads are thoroughly mixed at all times. Vortex if needed.
3. Add 143µl of QMN beads to the tubes containing the samples. Vortex for 3 seconds and briefly centrifuge.
4. Incubate at room temperature for 15 minutes.
5. Place samples into magnetic stand until the beads have fully migrated.
6. Discard the supernatant and keep the beads. Ensure as much supernatant is removed as possible with 10µl tips.
7. With the beads on the magnetic stand, add 200µl of the fresh 80% ethanol to each sample.
    - To ensure the beads are properly washed, rotate the tube THREE times so that the beads flow through the ethanol as shown in the **Figure S1**.

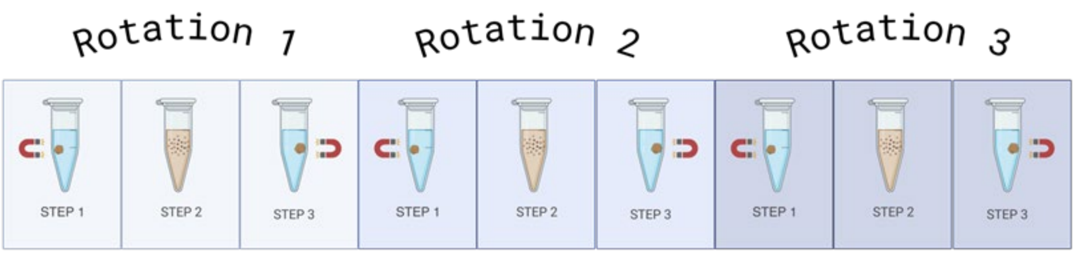

Figure S1. Rotate the Eppendorf tubes three times by 180° at each wash step to ensure complete washing of the magnetic beads.

8. Remove ethanol as quickly as possible, and repeat the wash step in step 7, ensuring you rotate the tubes THREE times once more.
9. Once complete, all traces of ethanol wash must be removed. To do so:
    - Remove ethanol with 200µl pipette
    - Remove from the magnetic stand, and centrifuge
    - Place back on the magnetic stand and carefully remove any excess ethanol wash with a 10µl pipette (note: try not to touch the walls of the tube with the tip).
10. With the beads still on the magnetic stand, leave to air dry with the lid open for 20 minutes (note: be sure to check the sides of the tube for any ethanol wash, even the smallest amount will interfere with the final yield and quality).

- The beads will form microcracks when dry and no residue is on the walls of the tube, as shown in **Figure S2**.

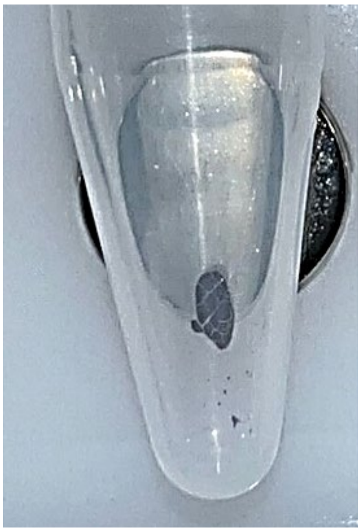

Figure S2. After effective drying, the presence of microcracks will appear in the beads, please also ensure there is no ethanol remaining on the sides of tubes.

11. Once the beads are fully dry, elute the cleaned cDNA with 17µl of nuclease free water. Mix by pipetting, vortex and briefly centrifuge.
12. Incubate the samples for 2 minutes at room temperature.
13. Return to the magnetic stand until the beads have fully migrated.
14. Transfer 15µl of the eluted cDNA to a new 96 well plate and seal.

The cleaned cDNA can be stored at -30°c to -15°c

End of Day 1

Supplementary Materials S7 **Library Amplification**

- The 15µl from the cDNA cleanup protocol is the starting material.
- Setup reactions on ice.
- Do not vortex any reagents.
- Ensure all reagents are added in the order stated in reaction mix tables.

1. Thaw the NGS library buffer and UDI Index plate. Mix by flicking and briefly centrifuge.
2. Remove the HotStarTaq DNA polymerase from the freezer, keep on ice and return to freezer immediately after use.
3. Pierce wells required one by one on the UDI index plate to assign each sample a unique index, make note of which UDI is assigned to each sample.

4. On ice, prepare the reaction mix below into the 96 well plate:

Reagent	***Volume (µl)***
cDNA cleanup product (already in well)	15
QIAseq miRNA library buffer	10
HotStarTaq DNA polymerase	1.5
Nuclease free water	21.6
UDI index	2
Total Volume	***50***

Table S8. Preparation of the cDNA reaction mix. Please add in the order stated.

5. Seal plate and briefly centrifuge. Then mix by pipetting 20 times, seal and briefly centrifuge again.
6. Programme the thermocycler as follows:

Step	**Time**	**Temperature (ºC)**
Hold	15 min	95
	3 Step Cycling	
Denaturation	15s	95
Annealing	30s	60
Extension	15s	72
Hold	2 min	72
Hold	∞*	4

Number of cycles = 24

Table S9. Thermocycler incubation for PCR amplification of the miRNA libraries.

***for at least 10 minutes at 4ºC**

7. Briefly centrifuge the plate, and transfer into labelled 1.5ml tubes.
8. Add 47µl of QMN beads to each sample (ensure the QMN beads are thoroughly mixed by vortexing the tubes).
9. Vortex the sample and the QMN beads for 3 seconds and briefly centrifuge.
10. Incubate the samples for 15 minutes at room temperature.
11. Place on the magnetic stand until the beads have fully migrated.
12. Keep 92µl of the supernatant and transfer to new labelled 1.5ml tubes.
13. To the supernatant, add 83µl of QMN beads. Vortex for 3 seconds and briefly centrifuge.
14. Incubate samples for 15 minutes.
15. Place the samples on the magnetic stand until the beads have fully migrated.

16. Discard as much of the supernatant as possible.
17. Wash the beads with FRESH 80% ethanol
    - Ensure the same level of extensive washing as in the cDNA clean up wash steps (step 7-8).
18. Repeat the wash step in step 17. Ensuring as much ethanol is removed from the tube as possible.
    - Ensure the same level of removal of the ethanol wash as in the cDNA clean up.
19. With the beads still on the magnetic stand, air dry the beads for 20 minutes or until completely dry.
20. Elute the library with 17μl of nuclease free water. Mix by pipetting, vortex and then briefly centrifuge.
21. Incubate the samples for 2 minutes at room temperature.
22. Place on the magnetic stand until the beads have fully migrated.
23. Transfer 15μl of the eluted miRNA library to new labelled tubes.

This is the miRNA sequencing library, which can be stored at -30ºC to -15ºC.

End of Day 2

Proceed to quality control of miRNA library.
